# Co-Isolation of Cytokines and Exosomes: Implications for Immunomodulation Studies

**DOI:** 10.3389/fimmu.2021.638111

**Published:** 2021-04-19

**Authors:** Shawna Benjamin-Davalos, Marina Koroleva, Cheryl L. Allen, Marc S. Ernstoff, Shin La Shu

**Affiliations:** ^1^ Department of Medicine, Roswell Park Comprehensive Cancer Center, Buffalo, NY, United States; ^2^ ImmunoOncology Branch, Developmental Therapeutics Program, Division of Cancer Treatment and Diagnosis, National Cancer Institute, Frederick, MD, United States

**Keywords:** cytokine co-isolation, exosome-associated cytokines, exosome isolation, tumor microenvironment, melanoma, soluble cytokines, REIUS

## Abstract

Exosomes play a vital role in intercellular communication and their immunomodulatory potential have become an important focus in cancer research. Various methods have been developed for the isolation although each method differs in the number and purity of exosomes they yield. In melanoma, tumor-derived exosomes drive immunosuppression within the tumor microenvironment. The co-elution of exosomes and soluble factors such as cytokines during isolation, however, make it difficult to ascertain the contribution of exosome cargo, as soluble cytokines are equally capable of immune suppression. In this review we will expound upon the biological relevance that exosome-associated cytokines possess. Furthermore, we discuss the technical challenges that arise during exosome isolation and what this means for further studies into the TME and *in vivo* work.

## Introduction

The tumor microenvironment (TME) has been the mainstay of tumor biology research for over 20 years. Cross talk between the immune system and the TME promotes immunosuppression, proangiogenic tendencies, and the inhibition of tumor cell death. Cytokines facilitate intercellular communication between immune cells as well as to other cells within the tumor microenvironment. Therefore, most efforts have focused on the exploitation of the immune system to eliminate cancer using cytokines. Multiple approaches including immunotherapy and cytokine therapy have been used as potential strategies to treat cancer.

Cytokines are categorized as immune-modulating, soluble factors that are less than 30kDa in size and include chemokines, interferons, interleukins, lymphokines and the tumor necrosis factor family of proteins (1). These molecules function to integrate signals derived from various cell types and to control the growth and activity of their target cell. Cytokines affect almost every biological process, and their downstream effects underlie diseases such as Alzheimer’s, autoimmunity, and cancer (2–4).

Cells also release lipid bound vesicles into the extracellular space. These extracellular vesicles (EVs) carry a variety of cargo that includes lipids, proteins and nucleic acids. EVs can be divided into three categories: microvesicles, apoptotic bodies, and exosomes, which differ in the method of biogenesis, release, size and content (5). A single protein cannot distinguish between these categories, although their differences in biogenesis contribute to the distinct proteomic profiles between classes. Microvesicles (MVs) are formed from the outward budding of the plasma membrane and range in size from 100 nm to 1 µm in diameter. The exact method of MV formation is not well characterized, but they contain many cytosolic and plasma membrane proteins. MVs are mediators of intercellular communication as their cargo is taken up by recipient cells and can subsequently alter function (6). Apoptotic bodies range in size from 50-5 µm and are released by dying cells *via* blebbing, a hallmark of cell death and the consequence of the disintegration of the cytoskeleton (7). Distinct from other types of EVs, apoptotic bodies contain chromatin, and intact organelles (8).

**Figure 1:**
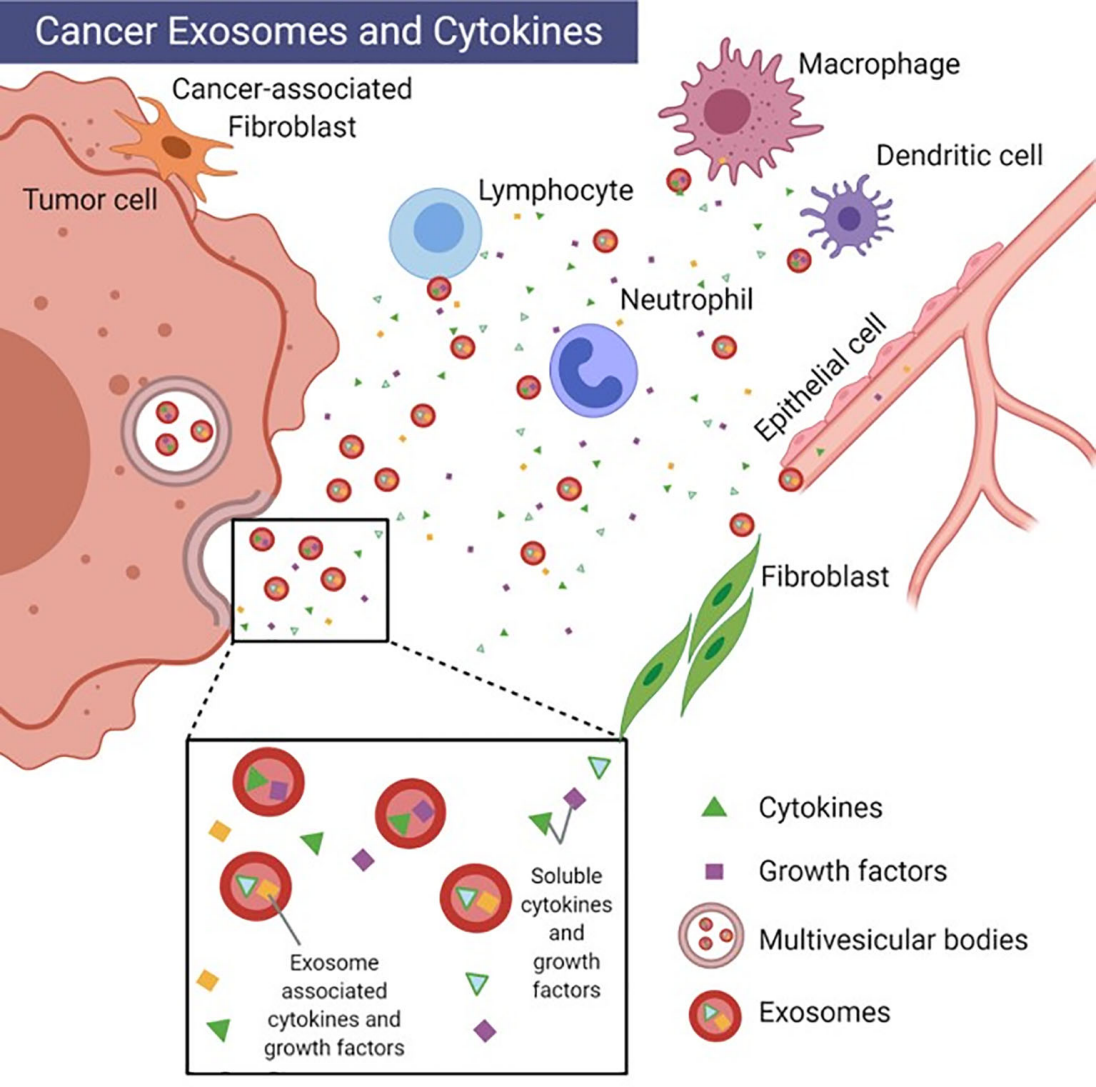
Cancer exosomes and cytokines. Depiction of uni-directional intercellular communication between tumor cell exosomes, immune cells and stromal cells. Image created with BioRender.com.

A subset of EVs ranging in size from 30-150 nm are exosomes which have been implicated in the progression of cancer through the trafficking of bioactive signals, either embedded in their membranes or packaged as payloads. Exosomes are formed from the release of multivesicular bodies into the extracellular space where they are either taken up by cells locally or travel through the bloodstream to more distal sites. Exosomes contain a variety of cytoplasmic proteins as well as nucleic acids (DNA, RNA) and various lipid species capable of translating immunomodulatory effects to cells (9). The secretion of EV-associated cytokines provides an additional mechanism by which cells can maintain specificity and integrity of signaling to distal cells (10). Cytokines possess a high affinity for their corresponding receptors, and thus are effective at concentrations in the picomolar range. Because of this, their secretion is tightly regulated (2). It is now recognized that they may also be packaged into exosomes as exosome-associated cytokines (EACs) (**Figure 1**) (10). Exosomes are thought to enhance the specificity of cytokine signals through the presence of receptors such as MHCs, tetraspanins and lactadherins in the exosome membrane, which are important for targeting other cell types (11). The release of both free and exosome-associated cytokines can potentially complicate *in vitro* work. Nevertheless, the ability to package a variety of critically bioactive molecules within a stable, lipid membrane-bound vesicle makes exosomes appealing to study as vehicles for therapeutics (12).

With the increasing potential for the use of exosomes clinically, it has become important to optimize exosome isolation methods to ensure maximum purity, yield and reproducibility. Due to the variety of exosome isolation methods utilized, the question arises as to whether current techniques can isolate exosomes devoid of cytokines and other soluble factors, which can confound the causative associations reported in literature. In this review, we will assess the biological role of EACs. Furthermore, we will discuss the technical challenges that preclude us from excluding soluble cytokines from exosome isolates and how this can complicate studies of the TME as well as other *in vivo* studies.

## Precedent for Membrane-Bound Cytokines

Multiple factors including cell origin, health status and environmental factors prior to exosome isolation can impact the variety and quantity of secreted cytokines present in exosome samples (13–15). Estrogen receptor (ER)-negative breast cancer cell lines secrete larger quantities of IL-6 compared to ER-positive cell lines (16). Nutrient deprivation of lung carcinoma (A549) cells for 24 hours showed an increase in the secretion of specific proinflammatory cytokines such as IL-6, IL-8 and chemokine CXCL1 compared to nutrient enriched cells (17). Moreover, subjects exposed to hypoxic conditions within a hypoxic chamber secreted more proinflammatory cytokines than those in standard conditions (18).

To decrease a cytokines’ effective range and thereby maintain specificity, many cytokines also function as a membrane-bound protein. Several cytokines and their receptors including IL-1, M-CSF, TGFβ, and TNFα have been reported in other studies to exist in both soluble and membrane-bound forms, both which are biologically active (19–23). Most notably, proinflammatory cytokine TNFα was found to also exist in a transmembrane form where it functions as a precursor to soluble TNFα and is able to exert its own cytotoxic activity by acting as a ligand for TNF receptor 2 (TNF-R2) (21, 24).

This logic can apply to EVs as well (25). Cytokines may be prepackaged into vesicles as a means of protecting these signals from degradation; however, controversy exists surrounding whether cytokines are displayed on the exosome surface or packaged into the payload. Additionally, the presence of surface-displayed cytokines can confound analyses for soluble cytokines. Fitzgerald et al. showed that EACs are either membrane-bound or EV-encapsulated (26). While encapsulated cytokines can be detected by lysing EVs prior to standard detection assays such as ELISA, the detection of membrane-bound cytokines using traditional cytokine detection methods is not as clear. Nine cytokines- IL-6, IL-8, IL-13, IL-16, IP-10, MCP-1, MIP-1α, MIP-1β and MIP-3α were found more often in soluble form (26). However, we have recently shown that the exosome isolation method used can impact the presence of soluble cytokines coeluted with exosomes. When corrected for the number of isolated exosomes, the **R**apid **E**xosome **I**solation using **U**ltrafiltration and **S**ize-exclusion chromatography (REIUS) method, led to an 836-fold reduction in 13 cytokines including IL-10 compared to the more commonly used ultracentrifugation (UC) isolation method, suggesting that the cytokines detected may not be physically associated with exosomes (27). The absence of data explicitly showing the association of cytokines to either the EV-membrane or the payload proves to be a major limitation for a number of studies, including our own. Exosomes do demonstrate the capacity to contain membrane-bound cytokines: rheumatoid arthritis synovial fibroblasts (RASF)-derived exosomes induced cytotoxicity in L929 cells (TNF-α-sensitized cell line) upon incubation for 24 hours (28). Colloidal gold immunostaining using electron microscopy (EM), was used to confirm the presence of membrane-bound TNF-α. Other cytokines with a known membrane-bound form in the originating cell could likely be packaged into the EV membrane. Zhang et al. reported that renal cancer (RC-2) cells transfected with engineered glycolipid-anchor-IL-12 packaged this membrane-bound form into exosomes, which reversed tumor exosome-mediated inhibition of T cell activity (29). Again, although inferred, they did not show direct GPI-IL-12 association with exosomes by EM.

Fitzgerald et al. also showed that whether a cytokine will be secreted or packaged into exosomes is dependent upon the biological system and nature of the stimulus (26). Placental villous explants preferentially secreted a subset of cytokines (IL-6, IL-8, IL-15, GRO-α, IP-10, MCP-1, MIP-1β, MIP-3α, and RANTES) in soluble form, whereas T cells and monocytes in culture produced cytokines in an EV-associated form. Other tissues and body fluids included in the study presented a more even distribution of soluble and EACs. The distribution of cytokines between molecular forms was dramatically changed among activated monocytes stimulated with either lipopolysaccharide (LPS) or Toll-like receptor (TLR3) agonist Poly I:C. Poly I:C-activated monocytes shifted toward the production of more soluble cytokines, demonstrating that the form of expression is not the property of a particular cytokine. How these signals are perceived by target cells and whether or not a difference in the secreted form of cytokines elicits different functions in target cells remains to be addressed. Rana et al. has shown that poly I:C-stimulated keratinocytes could secrete both soluble and EV-associated forms of IL-36γ implying that separate, regulated signaling pathways exist for cytokine secretion (30). The loading of these cytokines into vesicles may involve the function of chaperone proteins as HSP90 was found to be required for the translocation of IL-1β onto vesicle intermediates (31). Differences in biological function have also been demonstrated for soluble versus membrane-bound cytokine receptors. Specifically, soluble IL-6 receptor in complex with IL-6, elicits pro-inflammatory trans-signaling whereas the binding of IL-6 to membrane-bound IL-6R promotes anti-inflammatory downstream effects (32, 33).

## Quantitative Look at Cytokine Presence in Exosome Isolates

There is no strong evidence to suggest that exosomes can be isolated with the complete exclusion of cytokines. In 2016, Gardiner et al. showed that 81% of studies used UC over other isolation methods (34). UC separates EVs using centrifugal force and thus at high speeds, EVs are pelleted while particles that are less dense remain in the supernatant (35). As research on EVs has expanded over the years, additional isolation techniques have been developed. These techniques, which include ultrafiltration (UF), and size exclusion chromatography (SEC), involve separating exosomes based on molecular weight or size/diameter. In addition, modified methods such as REIUS, affinity-capture based methods, exosome precipitation and microfluidic-based techniques exist as well- each comes with its own advantages and disadvantages as an increase in exosome yield does not necessarily correlate with enhanced exosome purity (**Table 1**). Immunoaffinity-capture- based techniques are highly specific and result in pure isolations, but is highly dependent on surface marker specificity, which may cause the isolation of a subset of exosomes instead of the full heterogenous composition. As with any antibody-based purification method it is highly sensitive to the concentration of antigen (e.g. exosomes) and this may cause low yield of exosome isolation. Large-scale analyses of heterogeneous exosome populations may become biased toward certain subsets of exosomes if captured on enriched but not exclusive, exosome markers using immunoaffinity capture such as TIM4 and tetraspanins CD63, CD9 and CD81 (39). Phosphatidylserine present on the surface of exosomes has also been proposed as a method for detecting cancer exosomes (45). Exosomal precipitation methods utilize water-excluding polymers like polyethylene glycol (PEG) in popular commercial products such as ExoQuick (36). These polymers bind water molecules, while less soluble molecules are isolated by centrifugation. This method yields exosomes of low purity and number as contaminants are co-isolated along with EVs (42). Microfluidics-based techniques rapidly isolate exosomes based on their physical and biochemical properties and can address issues of purity through isolating subsets of exosomes similar to affinity-based methods, or by isolating exosomes based on size alone (43, 44). The use of these methods to mitigate cytokine contamination requires further investigation.

**Table 1:** Summary of existing exosome isolation methods.

Isolation method	Principle	Advantages	Disadvantages	Purity	Ref.
Ultracentrifugation (UC)	Size Density	Low cost Moderate yield	Time-consuming Labor intensive Expensive equipment required Requires large amount of starting material Propensity for forming aggregates	Moderate	(27, 35)
Ultrafiltration (UF)	Size Molecular weight	Quick Low cost	Time consuming High contamination Low specificity Low yield Prone to aggregation	Low	(11, 36)
Size exclusion chromatography (SEC)	Size Shape Molecular weight	Quick High yield	Low efficiency	Moderate	(37)
Rapid exosome isolation using ultrafiltration and size exclusion chromatography (REIUS)	Size Molecular weight	High yield	Requires multiple types of starting materials	High	(27, 38)
Affinity-capture methods (Immunoaffinity)	Antibody binding	Highly specific	Expensive Time consuming Isolate specific subsets of exosomes Cannot use for downstream assays Low yield	High	(39–41)
Exosome precipitation methods (PEG)	Solubility Surface charge	High yield Low cost Requires little starting material	Low specificity	Low	(36, 42)
Microfluidics	Size Density Immunoaffinity	High specificity	Time consuming High contamination Low specificity Low yield Prone to aggregation Retention of PEG in isolate	High	(43, 44)

UC, ultracentrifugation; UF, ultrafiltration; SEC, size exclusion chromatography; REIUS, Rapid exosome isolation using ultrafiltration and size exclusion chromatography; PEG, polyethylene glycol.

Shu et al. compared the soluble cytokine levels present in exosome isolations using REIUS, UC, UF, and SEC methods and found that exosomes isolated from the supernatant of two melanoma cell lines had varying cytokine levels depending on the isolation method used (26). All methods led to a decrease in the concentration of cytokines detected in exosome isolates compared to the original supernatants, however the REIUS method had the greatest impact in reducing the presence of soluble cytokines. REIUS isolation reduced cytokine concentrations by several logs in comparison to UC and outperformed the use of SEC alone, decreasing IL-10 levels by more than 8-fold to 97 pg/ml versus 814 pg/ml. This trend was the same for UF: 96.86 pg/ml of IL-10 was detected in the REIUS-isolated sample versus 44,450 pg/ml in the UF flow-through, demonstrating that the UF step is critical to reducing the concentration of soluble cytokines. IL-8 and IL-10 were present in high concentrations (cell type-dependent) but overall reduction in concentration was seen for all 13 cytokines tested thus concluding that while REIUS cannot remove all soluble cytokines, they are reduced to a greater extent when compared to other exosome isolation methods, ensuring higher purity without sacrificing exosome yield. The use of additional methods to mitigate soluble cytokine presence requires further investigation.

## The Effect of Cytokine Co-Isolation on *In Vivo* Studies and the TME

The inability to completely exclude cytokines from exosome isolates poses many challenges for downstream applications. Cytokines bear strong affinities for their corresponding receptor. They are also capable of eliciting proliferative and differentiative effects in specific cells among other functions, and thus have been used extensively as a cancer therapeutic. Proinflammatory cytokines such as IFNα, IL-2, IL-10, IL-12, IL-15 and GM-CSF have been tested for anti-tumor effects with varying success (46, 47). IFNα and IL-2 were the first cytokines to demonstrate antitumor effects *in vivo*; this observation led to the development of cytokines as anti-tumor monotherapies. Their use in clinical trials, however, has largely been terminated because of their level of toxicity, particularly at higher doses. High dose IL-2 administered to patients with metastatic melanoma had an overall response rate of 16%, but frequently led to the release of other proinflammatory cytokines causing capillary leak syndrome, flu-like symptoms and hypotension (48). In the current time, the expanded use of CAR T cells, which also elicits a clinical cytokine release syndrome, will also impact EV isolation methods. The co-elution of cytokines is relevant to those isolating exosomes, particularly when concentrating body fluids using ultrafiltration (UF), which will ultimately increase the concentration of co-isolated cytokines and could lead to toxicity.

Much work has been conducted to elucidate the role of tumor-derived exosomes within the TME. Cytokines such as TNFα, TGFβ, CSF-1, CCL2, CCL3, CCL5 and IL-8 can induce myeloid cell proliferation and promote immunosuppression within the TME. Human melanoma exosomes are also immunosuppressive. Exosomes expressing the checkpoint inhibitor programmed death ligand 1 (PD-L1), are released by melanoma, driving immunosuppression (49). Murine melanoma-derived exosomes were shown to increase proliferation and inhibit cell death of melanoma tumor in mice (50). In addition, HMEX induced cell death and inhibited proliferation in CD8+ T cells while downregulating NGK2D expression in natural killer (NK) cells (51). We have shown that HMEX exosomes are co-isolated with high concentrations of IL-8. The presence of soluble cytokines within exosome isolates cytokines can diminish or amplify the effects of exosomes, leading to erroneous conclusions. Changes in the functionality of exosomes purported to be immunosuppressive for instance, may indicate the presence of soluble cytokines in exosome isolates but improving upon purification methods in order to reduce the presence of soluble cytokines should not interfere with the behavior of the derived exosomes. Moreover, proinflammatory cytokines in exosome isolates can trigger inflammation, which can be damaging or even fatal.

EACs cannot be excluded from eliciting toxic effects. The stability and high biocompatibility of exosomes have made them an attractive vehicle for chemotherapeutics and other drugs. As such, research efforts have focused on determining which cell types are the most ideal source of exosomes. As exosomes embody a similar molecular profile to the originating cell, retention of the lipid and surface protein profile may prove important for proper exosome function (52, 53). Exosomes derived from melanoma tumor have been shown to be immunosuppressive through various mechanisms, which include the enhancement of the production of myeloid derived suppressor cells (MDSCs) and the presence of immunosuppressive EACs (40). Conversely, TEX antigen presentation to DCs as well as pro-inflammatory EACs may exacerbate the patient’s condition, illustrating the importance of choosing the proper cell-derived exosomes for therapy (54). To combat this issue, plant exosomes mainly derived from fruit and milk are currently being explored as an alternative as they are equally biocompatible and cheaper to produce. Exosomes from grapes did not produce any cytotoxic effects when administered orally to mice and may prove advantageous for administering treatments without unintentional effects (55). The role of using autologous derived human exosomes leveraging their inherent biology has yet to be explored as a therapeutic. However, care must still be taken to ensure that there is minimal presence of other unwanted soluble factors in the exosome product.

## Discussion

Current technical limitations related to the isolation method prevent us from completely removing soluble factors from exosome isolates, making it difficult to distinguish whether downstream effects are due to exosome function or that of the co-isolated cytokines. Moreover, they also prevent us from fully distinguishing between exosomes and other EVs. For instance, microvesicles bud off from the plasma membrane as opposed to being secreted into the extracellular space. As they overlap in size with exosomes and share common surface markers and cargo, they are commonly co-eluted with exosomes. These limitations prove detrimental to the understanding of the intricacies of the TME, particularly the contribution of exosomes in the maintenance of the TME.

The TME consists of tumor cells, vascular cells, fibroblasts and immune cells and the subsequent interaction of all these cell types contributes to the progression of cancer. HMEX are immunosuppressive and contain functional EACs (56, 57). As tumor cells secrete more exosomes compared to healthy cells, it is possible that the enhanced secretion seen in melanoma cells is a method of providing a large amount of stable, long range signals necessary for establishing a pre-metastatic niche (58). Because exosome production is a conserved process, almost all cell types produce and secrete exosomes.

**Figure 2:**
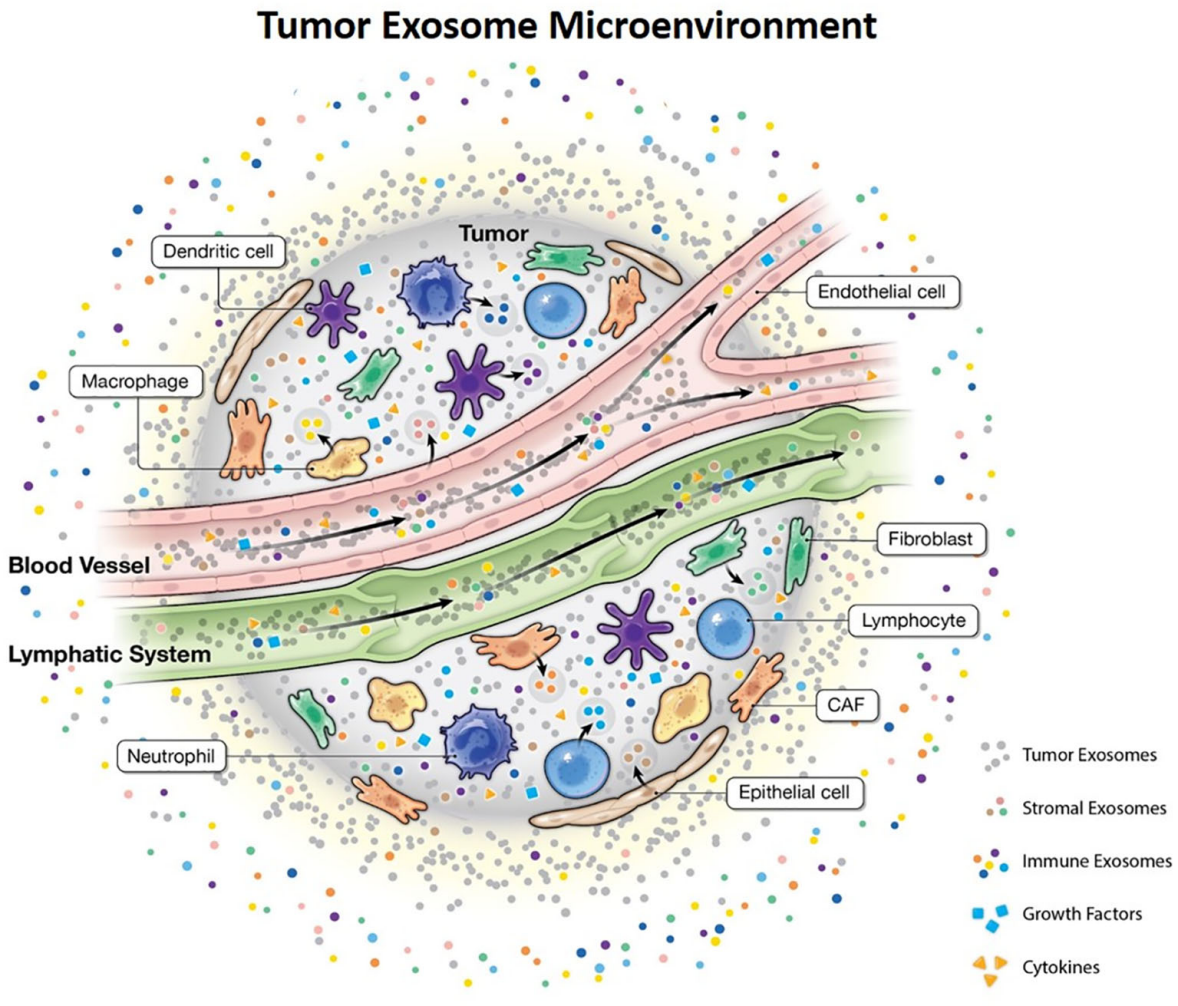
Tumor exosome microenvironment. The orchestrated interaction between various cell types, exosomes and soluble factors.

Exosomes contain specific protein markers corresponding to the originating cell type. Therefore, it would be hard to decipher a mixture of exosomes derived from various cell types from patient blood, which theoretically can serve as a representation of the TME (59). Sharma et al. demonstrated that the separation of melanoma-derived exosomes from non-tumor exosomes in patient plasma was possible using an antibody against a specific epitope of tumor antigen CSPG4, which is expressed in melanoma cells, but not in normal cells. Ultimately, this alludes to the heterogeneity of exosome populations (60). The existence of subpopulations of exosomes that differ in their proteomic and RNA profiles were found to have differential effects on target cells. Ideally, deciphering the TME would be much simpler if signaling between cells was solely unidirectional however, it is more plausible that bi- and multi-directional communication takes place between the tumor and resident cells of the TME as exosomes display a variety of ligands and receptors allowing for their interaction with multiple cell types. This coordinated network of interactions between cells and signals in the form of secreted exosomes, growth factors and cytokines, termed the ‘tumor exosome microenvironment’ (TExME), underlies tumor progression and deciphering this network may help to predict patient prognosis (**Figure 2**).

The presence of EACs, specifically those within the payload of exosomes, is strongly supported by the literature. Numerous cytokines that include IL-2, IL-4, IL-10, IL-18, and IL-33, TNF-α, TGF-β, M-CSF were found to be preferentially enriched within exosomes (26, 59, 61–63). IL-10 in particular has been demonstrated to be exclusively in the exosome payload and is biologically active, inducing mitophagy in kidney tubular epithelial cells (64). Soluble cytokines that have been released extracellularly into supernatant or patient plasma have biological relevance, however, when focusing on the contribution of exosomes within this network of interactions, the inability to completely remove soluble cytokines from isolates makes it difficult to differentiate them from exosome surface-bound cytokines and only confounds our interpretation of these interactions. At best, combining more than one exosome isolation method may be the way forward for “fit-for-purpose” exosome purification to enhance immunomodulatory studies. For instance, immunoaffinity capture can be used after REIUS to complement the advantages of both methods. The combination of these methods should yield specific populations of exosomes of high purity.

Many factors including cell type and stimulus can affect the level of cytokine presence in an exosome sample as well as the form in which the cytokines are secreted, whether for local or more distant signaling. Understanding these interactions within the milieu that is the ‘tumor exosome microenvironment’ and further developing technologies to be able to tease them apart is critical to more targeted therapies.

## Author Contributions

SB-D: contributed to the general idea, wrote and edited the manuscript, and created figures. MK: contributed ideas to, compiled information for manuscript, and edited figures and manuscript. CA: contributed ideas to and edited manuscript. ME: provided expert opinion and input and edited manuscript. SL: conceived the general idea, created figures, and edited the manuscript. All authors contributed to the article and approved the submitted version.

## Funding

This work was supported by institutional funds provided by Roswell Park Comprehensive Cancer Center.

## Conflict of Interest

The authors declare that the research was conducted in the absence of any commercial or financial relationships that could be construed as a potential conflict of interest.
